# Prognostic value of lymph node ratio in laryngeal and hypopharyngeal squamous cell carcinoma: a systematic review and meta-analysis

**DOI:** 10.1186/s40463-020-00421-w

**Published:** 2020-05-29

**Authors:** Arikin Abdeyrim, Shizhi He, Yang Zhang, Gulbostan Mamtali, Aibadla Asla, Mirkamil Yusup, Jiang Liu

**Affiliations:** 1 Department of Otorhinolaryngology Head and Neck Surgery, People’s Hospital of Xinjiang Uygur Autonomous Region, 91 Tianchi Road, Tianshan, Ürümqi, Xinjiang, 830001 China; 2 Department of Otolaryngology-Head and Neck Surgery, Beijing Tongren Hospital, Capital Medical University, Beijing, China; 3 Department of Otorhinolaryngology, China-Japan Friendship Hospital, Beijing, China; 4 Department of Neurosurgery, China-Japan Friendship Hospital, No. 2, Yinghua East Street, Chaoyang District, Beijing, 100029 China

**Keywords:** Hypopharyngeal cancer, Laryngeal cancer, Lymph node ratio, Prognosis, Meta-analysis

## Abstract

**Background:**

Several recent studies have indicated that the lymph node ratio (LNR) is an independent prognostic factor for laryngeal and hypopharyngeal squamous cell carcinoma (LHSCC). The purpose of this paper is to assess the prognostic value of LNR and explore appropriate cutoff values by conducting a systematic review and meta-analysis.

**Methods:**

Pubmed, Embase (via Ovid), and Cochrane library were systematically searched for studies on the prognostic value of LNR in LHSCC up to October 31, 2019. Then, Literature review, data extraction, and quality assessment of eligible studies were performed by two independent reviewers back-to-back. Lastly, Stata 14.0 software was hired to conduct a meta-analysis.

**Results:**

A total of 445 articles were retrieved, and 13 studies published in English between 2013 and 2019 were included after the title/abstract and full-text screening. Among the 13 studies contributed to 4197 patients, seven studies were about hypopharyngeal squamous cell carcinoma (HPSCC), four studies about laryngeal squamous cell carcinoma (LSCC), and the remaining two studies about LHSCC. The meta-analysis results showed that shorter overall survival (OS) (HR 1.49; 95%CI: 1.18 to 1.88), disease-specific survival (DSS) (HR 1.66; 95%CI: 1.32 to 2.07) and disease-free survival (DFS) (HR 2.04; 95%CI: 1.54 to 2.71) were significantly correlated with a higher LNR in a random-effect model. The cutoff values of eligible studies were varied from 0.03 to 0.14, and the lowest significant LNR was 0.044.

**Conclusion:**

LNR is a valuable prognostic factor in the survival of LHSCC and may be used to improve the tumor staging systems, which, however, requires the solid support of more high-quality studies.

## Introduction

Treatment of laryngeal and hypopharyngeal cancer was frustrating due to its dysfunction and poor prognosis. Surgery is the standard treatment chemoradiotherapy alone has also shown efficacy in organ preservation trials, especially for advanced diseases [1]. However, some patients who have received surgery with adjuvant chemoradiotherapy after surgery, may still have a relapse. Therefore, it is of importance to improve outcomes via seeking valuable prognostic factors and identifying patients at high risk of recurrence.

Based on the tumor-node-metastasis (TNM) staging, the American Joint Committee on Cancer (AJCC) system [2] is the most commonly used staging system for laryngeal and hypopharyngeal cancers, which classified the status of lymph nodes of patients by their number, size, laterality, and extra nodal extension (ENE) [3]. Nevertheless, each factor’s independent impact on clinical outcome remains unclear.

Lymph node ratio (LNR) or lymph node density (LND) is calculated as the number of positive lymph nodes divided by lymph node yield (LNY) removed through neck dissection [4]. Several studies showed that LNR could be employed as a supplement to the TNM staging system for better assessment of survival for esophageal [5], colorectal [6, 7], gastric [8], and breast cancers [9]. In addition, two meta-analyses showed that LNR might be a significant prognostic indicator for head and neck malignancies [10] and oral [11] squamous cell carcinomas. Several recent studies [12–16] indicated that LNR was related to survival in patients with LHSCC, and reported on various cutoff points.

In this paper, a systematic review and meta-analysis were conducted to assess the prognostic value of LNR and explore appropriate cutoff values.

## Methods

The study was conducted following the Preferred Reporting Items for Systematic Review and Meta-Analyses (PRISMA) guidelines [17].

### Search strategy

We searched for the combination of free text words like “laryngeal,” “larynx,” “hypopharyngeal,” “hypopharynx,” “cancer?,” “carcinoma?,” “neoplasm?,” “lymph nodes,” “nodal,” “ratio,” and “density,” in the PubMed, Embase (via Ovid), and Cochrane library systematically to find articles on the prognostic value of LNR in LHSCC up to October 31, 2019. Medical Subject Headings (MeSH) were adopted to in combination with the Boolean operators “AND” or “OR.” The detailed retrieval strategy was shown in Tables S1, S2 and S3. Additionally, references cited in the articles were checked and found to be available.

### Study selection

We had access to all published articles which evaluated the prognostic values of LNR in LHSCC, and included articles that were published in English, studied LHSCC patients with positive lymph nodes, reported calculated LND or LNR, clarified the LNR-related outcomes, and applied multivariate regression analysis to analyze the relationship between LNR and outcomes. As previous studies showed that no significant difference was noted between LSCC and HPSCC in LNR [18], we combined LSCC with LSCC for analysis.

We excluded studies whose full text were unavailable, as well as studies that were published as abstracts, reviews and case reports and not focused on the association between laryngeal or hypopharyngeal cancer and LNR. For studies that may overlap in the included population, we preferred the high-quality ones.

The literature selection was carried out by two researchers (AA and HSZ) independently, and any disagreement was resolved through negotiation or seeking the help of third party.

The outcomes were overall OS, DSS and DFS. OS was defined as the period from surgery to the last follow-up or death [19–22]. DSS was referred to the period from the initial diagnosis to LHSCC-related or treatment-related death [16, 20–22]. DFS was defined as the period from the surgery to the last follow-up with no evidence of recurrence or distant metastasis [19, 21, 22].

### Data abstraction

Two researchers (AA and HSZ) independently extracted the following information: author, year of publication, region, type of study, population basic characteristics, tumor basic characteristics, tumor treatment, follow-up time, and outcomes. If they did not reach a consensus over conflicting results, a third reviewer would participate making a decision.

### Quality assessment of eligible studies

On referring to previously published meta-analyses [11, 23], we made the quality assessment of eligible studies based on the Reporting Recommendations for Tumor MARKer Prognostic Studies (REMARK) guidelines [24, 25]. The quality assessment covered the following eight domains: inclusion and exclusion criteria clarified, study design information, information of patient characteristics, information of tumor characteristics, LND or LNR measurements clarified, study outcomes defined clearly, follow-up time, and patient’s attrition clarified.

Scores for each study ranged from 0 to 8, with 8 representing the highest quality and 0 the lowest [11, 23].

### Statistical analysis

In order to assess the relationship between LNR and LHSCC, we calculated the pooled hazard ratio (HR) with a 95% confidence interval (CI). The lowest LNR value was extracted for analysis, while the original articles presented two more categories. Based on the 95% CI not coming across the null line, HR greater than 1 indicated poor prognosis in LHSCC patients with a higher LNR value. If two more studies reported an outcome, we performed data synthesis in a random-effect model. Then, we applied the Cochran Q-static and I^2^ tests to estimate heterogeneity between studies [26], and a *P*-value of Q-test less than 0.05 or an I^2^ value higher than 50% indicated large heterogeneity between studies. Subsequently, the subgroup analysis and the meta-regression analysis were conducted to explore the sources of heterogeneity. The potential publication bias of eligible studies was identified using Funnel plots, the Begg’s test, and the Egger’s test. We considered a *p*-value of less than 0.05 statistically significant. The trim and fill method was applied to adjust the results of pooled analysis in the case of publication bias, and all statistical tests were two-sided. Statistical software Stata Version 14.0 (StataCorp, College Station, TX, 2014) was employed in the review.

## Results

**Fig. 1:**
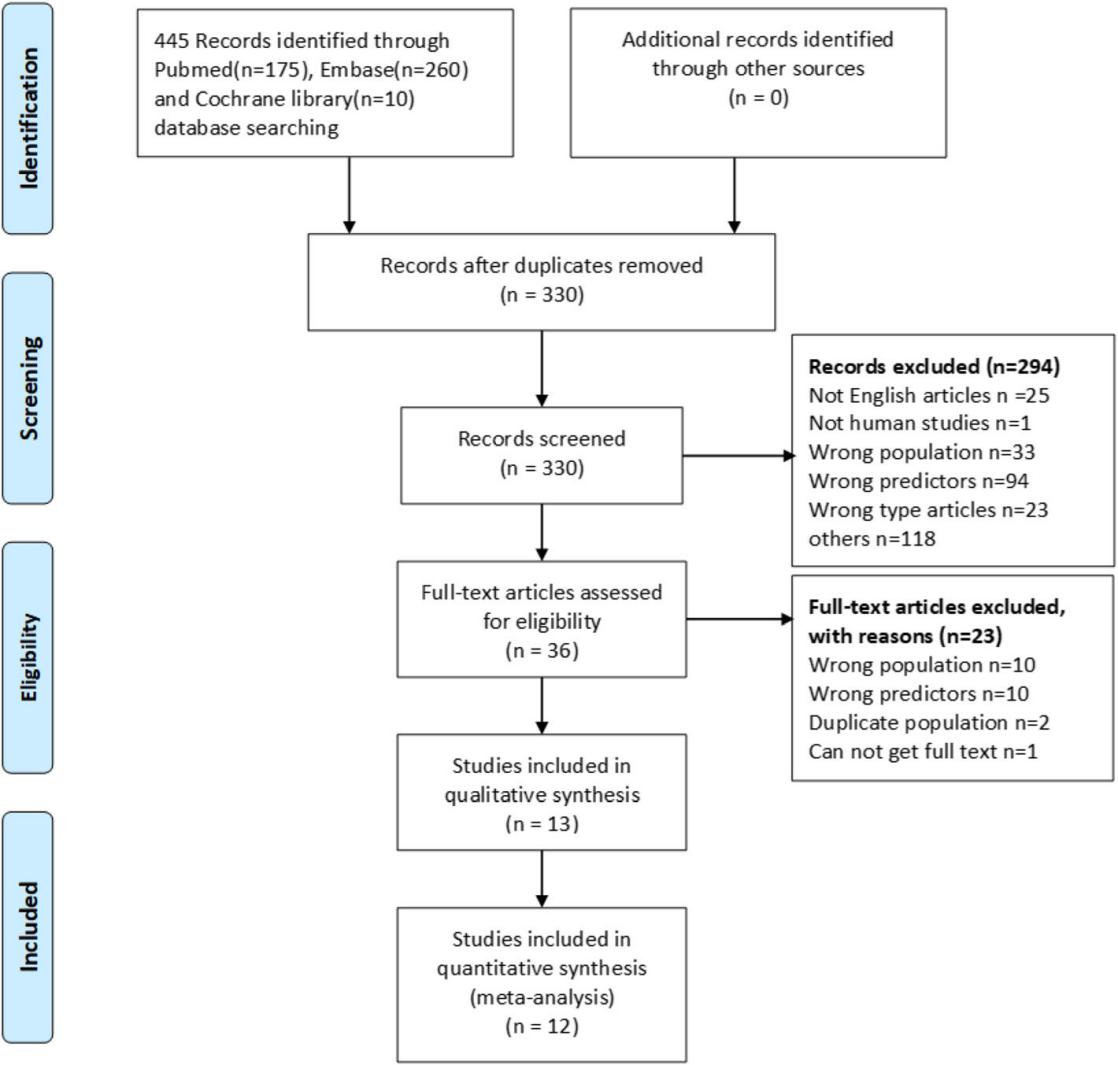
PRISMA literature screening flow diagram. Preferred Reporting Items for Systematic Review and Meta-Analyses (PRISMA) literature screening flow diagram

We retrieved 445 articles following the search strategy, among which 36 articles were eligible for the full-text screening process, and 13 articles [12–16, 18–22, 27–29] were included in the meta-analysis finally. Reasons for excluding studies in the full-text screening process were listed in Table S4. The PRISMA Flow diagram is shown in Fig. 1.

Among 13 articles contributed to 4197 patients (range, 46 to 1963; median, 105), seven studies were about HPSCC, four studies were about LSCC, and the remaining two studies were bout LHSCC. All studies were published in English between 2013 and 2019, with only one study contributed to 46 patients was designed as a prospective study, and the others as retrospective studies.

Seven [13–16, 21, 22, 29] and six studies [12, 18–20, 27, 28] were carried out in Eastern and Western, respectively. The mean or median follow-up time of included studies, median or mean lymph node yield, and median or mean number of positive lymph nodes range from 23 to 60 months, 20 to 60, and 1 to 4, respectively. The cutoff values of LNR calculated by different methods in eligible studies varied from 0.03 to 0.14, and the lowest significant LNR reported was 0.044. The basic features of the included studies, characteristics of patients and tumors are summarized in Tables 1 and 2.

**Table 1:** Main characteristics of the eligible studies

Study	Year	Region	Study period	Type of study	Population	Endpoints with HR	LNR cut-off	Method of LNR determination	Follow-up time (months)
Ye [22]	2018	China	2007 to 2016	retrospective	HPSCC	OS, DFS	<0.07,≥0.07	ROC	Median 33.1 (6.1 to 110.7)
Lo [15]	2017	Taipei, China	2001 to 2007	retrospective	HPSCC	OS, DFS, DSS	< 0.113, ≥0.113	Mean	Mean 51 (4 to 144)
Suzuki [21]	2016	Japan	2000 to 2015	prospective	HPSCC	OS, DFS	< 0.09, ≥0.09	Log-rank test	Mean ± SD 45.7 ± 40.6
Joo [14]	2015	Korea	1993 to 2014	retrospective	HPSCC	OS, DFS	< 0.055, ≥0.055	ROC	Mean 37 (1 to 151)
Hua [13]	2015	China	2000 to 2005	retrospective	HPSCC	OS	< 10, > 10%	NA	Median 48 (12 to 127)
Yu [29]	2013	China	1965 to 2008	retrospective	HPSCC	OS, DFS, DSS	≤0.14, > 0.14	LNR quartiles	Mean 41.8 (1 to 295)
Wang [27]	2013	SEER	1988 to 2008	retrospective	HPSCC	OS, DFS	0–0.05, 0.05–0.30, > 0.30	Log-rank test	Median 23 (0 to 238)
Imre [19]	2016	Turkey	2006 to 2014	retrospective	LSCC	OS, DFS	< 0.09, ≥0.09	Univariate Cox analysis	Mean 33.5 (6 to 94)
Ryu [16]	2015	Korea	2001 to 2010	retrospective	LSCC	DFS	≤0.044, > 0.044	ROC	Median 53.2 (2.1 to 147.1)
Wang [28]	2014	SEER	1988 to 2008	retrospective	LSCC	OS, DFS	0–0.09, 0.09–0.20, > 0.20	Log-rank test	NA
Künzel [20]	2015	Germany	1980 to 2010	retrospective	LSCC	DFS	< 0.09, > 0.09/< 0.09, > 0.09	ROC	Mean 53 (0.1 to 229)
Choi [12]	2019	Germany	2006 to 2016	retrospective	HPSCC/LSCC	OS, DFS, DSS	0.03	ROC	Median 60 (24 to 134)
Grasl [18]	2019	Austria	1994 to 2018	retrospective	HPSCC/LSCC	OS, DFS, DSS	≤0.07, > 0.07	ROC	Mean ± SD 55.6 ± 59.3

*HPSCC* hypopharyngeal squamous cell carcinoma, *LSCC* laryngeal squamous cell carcinoma, *OS* overall survival, *DSS* disease-specific survival, *DFS* disease-free survival, *ROC* receiver operating characteristic; NA: not available

**Table 2:** Patient and tumor characteristics of eligible studies

Author	Sample	Age	Male	Treatment	Type of Neck Dissection	T stage	N stage	Subsite	LNY	PN+
Ye [22]	93	Mean 56.9 (32 to 87)	98%	Surgery OR Surgery with (RT OR CRT)	Radical, Elective	T1 to T4	N0, N1, N2a/b/c, N3	Pyriform sinus, Posterior wall, Postcricoid region,	NA	Mean 3 (0 to 34)
Lo [15]	120	Mean 56 (35 to 89)	98%	Surgery OR Surgery with (RT OR CRT)	Bilateral, Unilateral	T1 to T4	N0, N1, N2a/b/c, N3	Pyriform sinus, Posterior pharyngeal wall, Postcricoid	NA	Mean 4
Suzuki [21]	46	Median 66	94%	Surgery OR Surgery with (RT OR CT OR CRT)	Extended radical, Modified Radical, Selective	T1 to T4	N1, N2	Priform sinus, Others	Median 59.5 (14 to 114)	Median 3 (1 to 15)
Joo [14]	105	Mean 61 (35 to 80)	97%	Surgery OR Surgery with (RT OR CRT)	Bilateral, Modified Radical, Radical, Selective	T1 to T4	N1 to N3	Pyriform sinus, Posterior pharyngeal wall, Postcricoid region	Median 54 (15 to 176)	Median 3 (1 to 31)
Hua [13]	81	Median 60 (36 to 80)	98%	Surgery OR Surgery with (RT OR CRT)	Bilateral, Unilateral	T1 to T4	N1 to N3	Pyriform sinus, Posterior wall, Postcricoid region	Median 47 (3 to 95)	Median 2 (0 to 17)
Yu [29]	279	Mean 57.8 (34 to 85)	93%	RT OR RT with (CRT OR ICT OR Surgery OR Surgery with CT)	Radical, Modified Radical, Selective	T1 to T4	N0 to N3	Pyriform sinus, Posterior wall, Postcricoid region	Median 20 (2 to 76)	Median 2.0 (0.5 to 22.0)
Wang [27]	916	Median 61 (35 to 93)	42%	Surgery OR Surgery with RT	NA	T1 to T4a/b, Tx	N1 to N3	Pyriform sinus, Posterior wall, Postcricoid region, Aryepiglottic fold, Overlapping lesion, Hypopharynx	Median 25 (1 to 90)	Median 2 (1 to 90)
Imre [19]	101	Mean 58.5 (41 to 79)	95%	Surgery OR Surgery with (RT OR CRT)	Radical, Modified Radical, Selective	T1 to T4	N1, N2a/b/c	Glottis, Supraglottis, Transglottis	Mean 41.8 (18 to 88)	Mean 2.9 (1 to 15)
Ryu [16]	71	Median 65 (35 to 79)	93%	Surgery OR Surgery with (RT OR CRT)	Bilateral, Modified Radical, Radical, Selective	T1 to T4	N0 to N2	Supraglottis, Glottis, Transglottis	Median 52 (11 to 153),	Median 4 (1 to 21)
Wang [28]	1963	NA	78%	Surgery OR Surgery with RT	NA	T1 to T4a/b, Tx	N1 to N3	Glottis, Supraglottis, Subglottis, Laryngeal cartilage, Overlapping lesion, Larynx	Median 28 (1 to 90)	Median 2 (1 to 90)
Künzel [20]	202	Median 59 (36 to 86)	93.1%	Surgery OR Surgery with (RT OR CRT)	Unilateral or bilateral modified radical	T1 to T4	N1, N2a/b/c	Glottic,Supraglottic,Unspecified site of origin within the larynx	Median 27 (10 to 88)	Median 2 (1 to 19)
Choi [12]	141	Median 65 (33 to 78)	93.6%	Surgery OR Surgery with (RT OR CRT)	Radical, Selective	T1 to T5	N1 to N3	Larynx, Hypopharynx	Median 60 (11 to 161)	Median 1 (0 to 21)
Grasl [18]	79	Median 60 (41.6 to 83)	88.6%	Surgery OR Surgery with (RT OR CRT/IRT)	Radical, Modified Radical, Selective	T1 to T4	N0, N1, N2a/b/c, N3a/b/c	Larynx, Hypopharynx	Median ± SD 48.0 ± 23.8	NA

*NA* Not available, *RT* Radiotherapy, *CT* Chemotherapy, *CRT* Chemoradiotherapy, *IRT* Radio-immune therapy, *LNY* Lymph node yield, *PN+* Positive lymph node number

The results of the quality assessment ranged from 4 to 7 (Table 3). Quality scores of 8 studies [14, 16, 18–22, 27] were more than 5, indicating that most of the studies were moderate quality.

**Table 3:** Quality assessment of eligible studies

Study	Inclusion and exclusion criteria	Prospective/Retrospective	Patient characteristics	Tumor characteristics	LNR measurements	Endpoint	Follow-up period	Patients unavailable for statistical analysis	Quality scale
Choi 2019 [12]	1	1	1	1	0	0	1	0	5
Ye 2018 [22]	1	1	1	1	0	1	1	1	7
Lo 2017 [15]	1	1	1	1	0	0	1	0	5
Suzuki 2016 [21]	1	1	1	1	0	1	1	0	6
Imre 2016 [19]	1	1	1	1	0	1	1	0	6
Ryu 2015 [16]	1	1	1	1	1	1	1	0	7
Kunzel 2015 [20]	1	1	1	1	1	1	1	0	7
Joo 2015 [14]	1	1	1	1	1	0	1	0	6
Hua 2015 [13]	0	1	1	1	0	0	1	0	4
Wang 2014 [28]	1	1	1	1	0	0	0	0	4
Yu 2013 [29]	1	1	1	1	0	0	1	0	5
Wang 2013 [27]	1	1	1	1	0	0	1	1	6
Grasl 2019 [18]	1	1	1	1	0	1	1	0	6

*LNR* Lymph node ratio

### Meta-analysis results

**Fig. 2:**
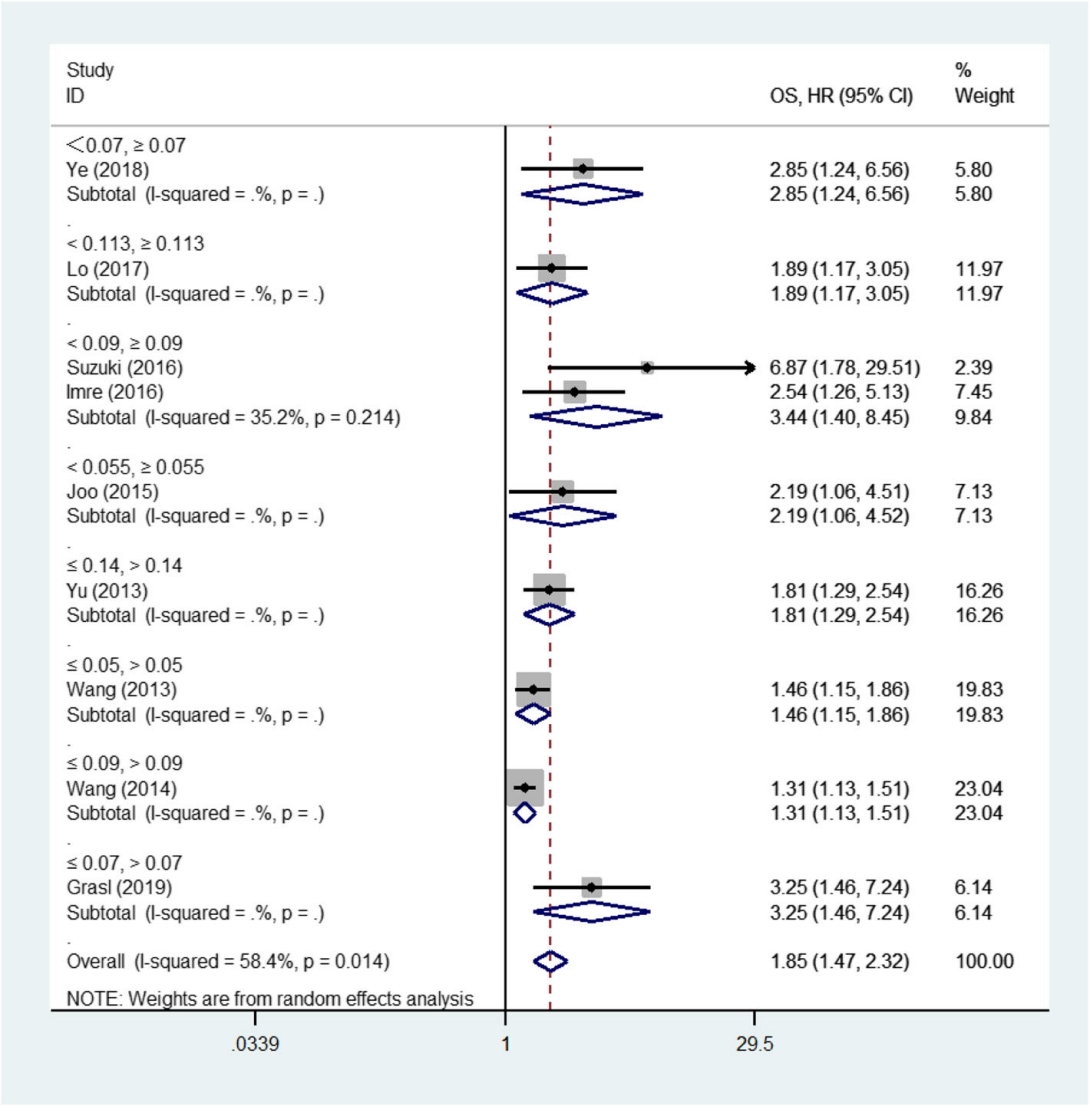
Forest plot of the meta-analysis regarding the overall survival (OS). Forest plot of the meta-analysis using the random-effect model in patients with LHSCC regarding OS. OS: Overall survival; HR: Hazard ratio; LHSCC: Laryngeal and hypopharyngeal squamous cell carcinoma

**Fig. 3:**
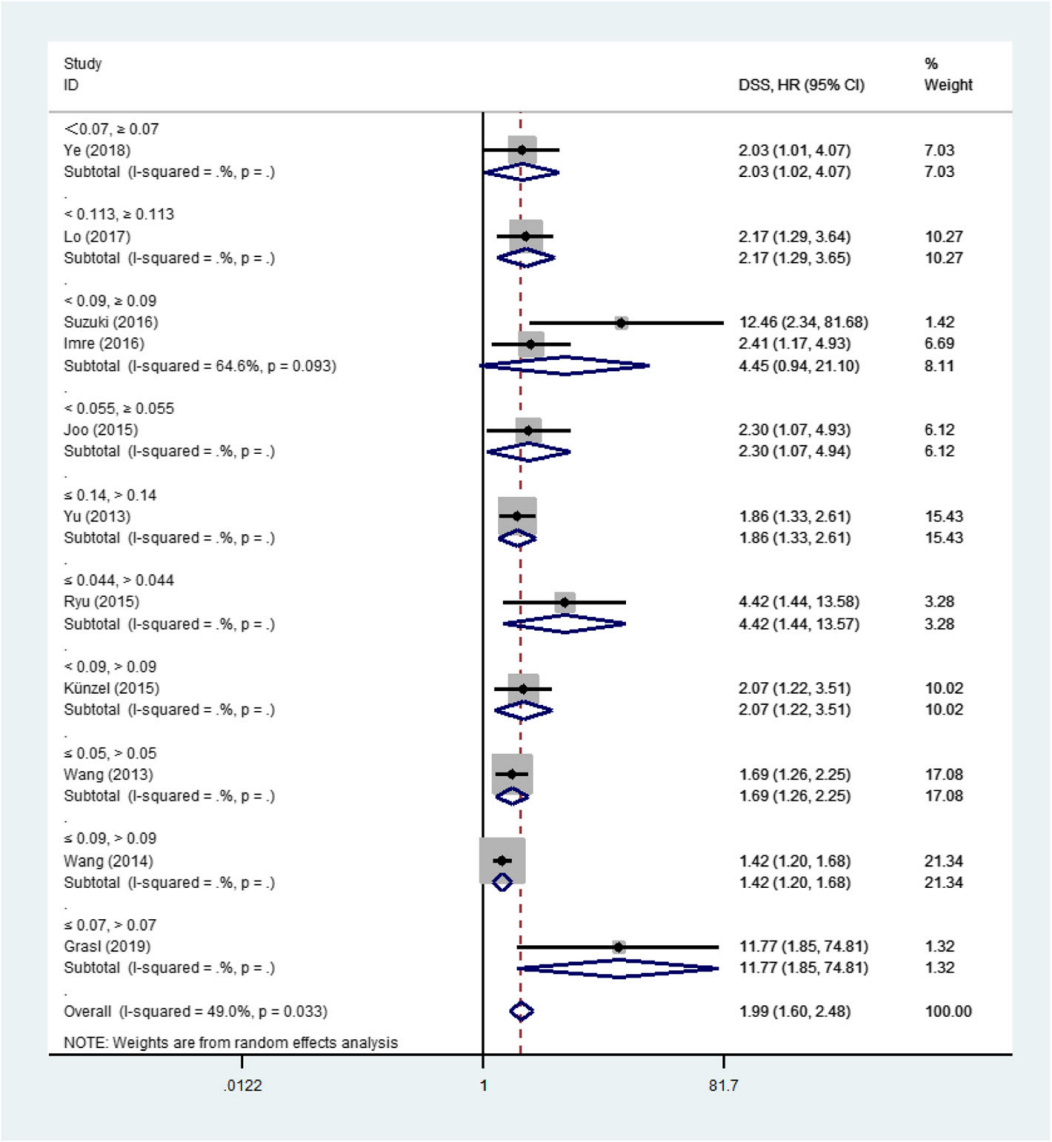
Forest plot of the meta-analysis regarding the disease-specific survival (DSS). Forest plot of the meta-analysis using the random-effect model in patients with LHSCC regarding DSS. DSS: Disease-specific survival; HR: Hazard ratio; LHSCC: Laryngeal and hypopharyngeal squamous cell carcinoma

**Fig. 4:**
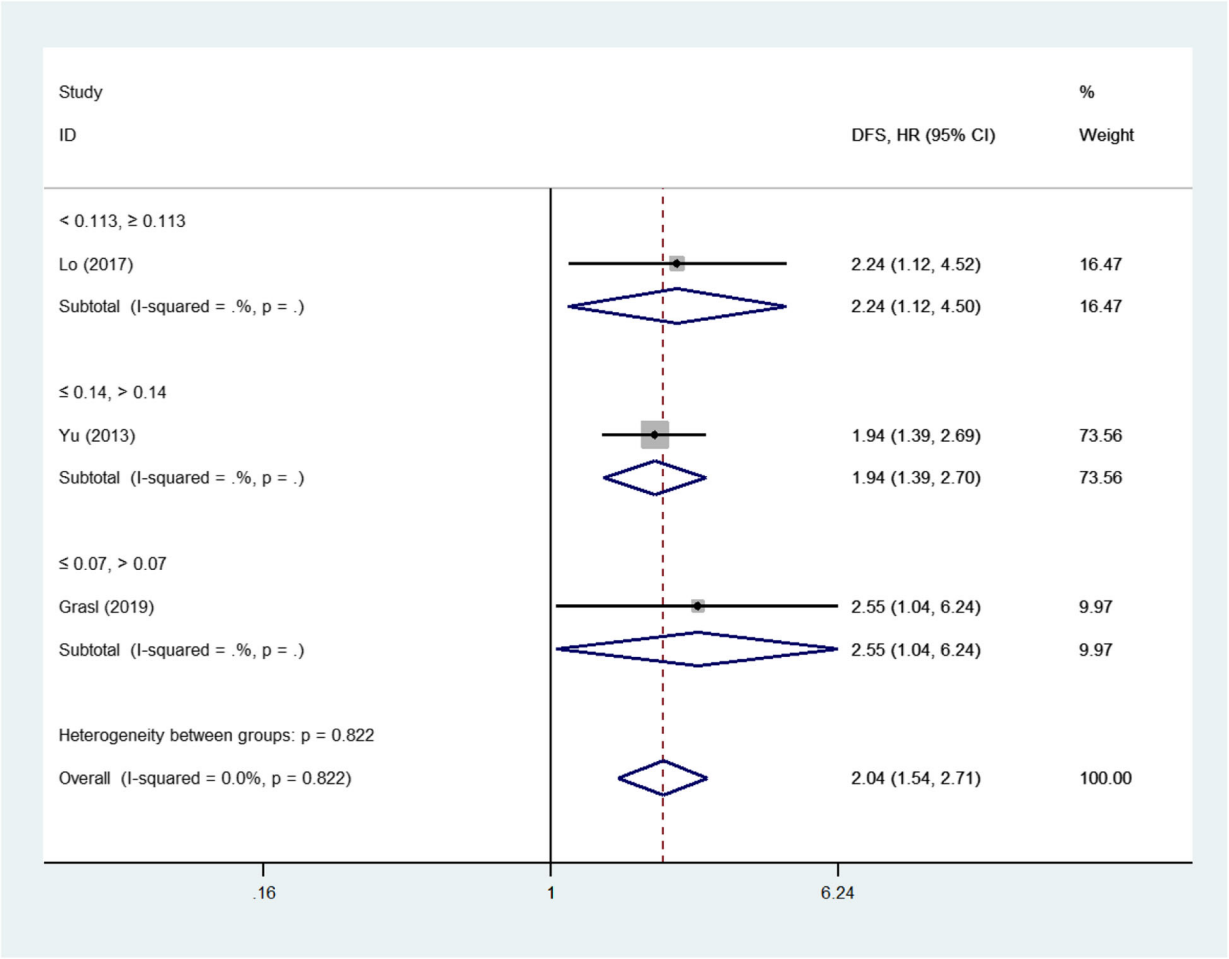
Forest plot of the meta-analysis regarding the disease-free survival (DFS). Forest plot of the meta-analysis using the fixed-effect model in patients with LHSCC regarding DFS. DFS: Disease-free survival; HR: Hazard ratio; LHSCC: Laryngeal and hypopharyngeal squamous cell carcinoma

Among the 13 eligible studies, 12 studies [12–15, 18–22, 27–29], 12 studies [12, 14–16, 18–22, 27–29] and 4 studies [12, 15, 18, 29] explored the association between LNR and OS, DSS, DFS outcomes respectively. The pooled analysis of studies reported LNR as the categorical variable showed the higher LNR values were associated with short OS (HR 1.85, 95%CI 1.47 to 2.32, 9 studies) in the random-effect model (I^2^ = 58.4%) (Fig. 2), short DSS (HR 1.99, 95%CI 1.60 to 2.48, 11 studies) in the random-effect model(I^2^ = 49.0%) (Fig. 3) and poor DFS (HR 2.04, 95%CI 1.54 to 2.71, 3 studies) in the fixed-effect model(I^2^ = 0.0%) (Fig. 4).

In addition, the meta-analysis results of studies [13, 27, 28] reported LNR as continuous variable showed that higher LNR values indicated poor OS (HR 1.88, 95%CI 1.63 to 2.17) and poor DSS (HR 2.33, 95%CI 1.97 to 2.74) in the fixed-effect model (I^2^ = 0.0%) (Fig. 3). Besides, one of the 13 studies [12] could not be quantitatively synthesized with other studies because its adjusted HR was unavailable. The univariate analysis suggested that LNR was a significant factor for OS, DSS and DFS outcomes (*P* < 0.05), while the multivariate analysis results did not favor LNR as an independent factor for OS, DSS and DFS outcomes.

The meta-regression analysis was carried out for OS outcome, but we were not able to identify factors that might affect inter-study heterogeneity. Besides, we conducted the subgroup meta-analysis to explore the sources of heterogeneity in OS outcome, but then when the studies divided into three subgroups by regions, the inter-study heterogeneity disappeared. Similarity, the pooled results suggested that high LNR were associated with short OS in Asian (HR 2.03, 95%CI 1.58 to 2.61, I^2^ = 2.6%), European (HR 2.83, 95%CI 1.67 to 4.79, I^2^ = 0.0%) and American (HR 1.35, 95%CI 1.19 to 1.52, I^2^ = 0.0%). Furthermore, the subgroup meta-analysis results based on quality scores of included studies also showed that a high LNR suggested poor survival, and the pooled HR values of subgroups with quality scores > 5 were 2.36 (95%CI, 1.57 to 3.54) for OS and 2.35 (95%CI, 1.70 to 3.25) for DSS. In addition, the subgroup analysis for laryngeal and hypopharyngeal cancers showed that increased LNR was associated with poor prognosis regardless of the tumor sites (Table 4).

**Table 4:** Subgroup analysis by tumor sites

Sites	OS	DSS	DFS
Hypopharyngeal cancer	HR 1.87, 95%CI 1.37 to 2.56, 6 studies	HR 1.89, 95%CI 1.47 to 2.42, 7 studies	HR 1.99, 95%CI 1.48 to 2.68, 3 studies
Laryngeal cancer	HR 1.74 95%CI (1.05 to 2.88), 2 studies	HR 2.11, 95%CI (1.34 to 3.34), 3 studies	NA

*OS* overall survival, *DSS* disease-specific survival, *DFS* disease-free survival, *NA* not available

### Publication bias

The Funnel plots of studies that reported OS (*p* < 0.01) and DSS (*p* < 0.01) in the meta-analysis were asymmetric. Moreover, quantitative evaluation results achieved by the Begg’s and Egger’s tests indicated that the publication bias was statistically significant. Five additional studies in OS (Fig. S1) and six in DSS (Fig. S2) that may be missed were filled with using the trim and fill method. After adjustment, the pooled HR value was 1.49 (95%CI, 1.18 to 1.88) for OS and 1.66 (95%CI, 1.33 to 2.07) DSS, indicating that the existing publication bias did not affect the final results.

### Sensitivity analysis

To ensure the robustness of the pooled adjusted HR values, we ruled out the adjusted HR value of the study of Yu et al. [29], which included population having received pre-operative radiation. We achieved the similar results, that is, an increased LNR value resulted in poor OS (HR: 1.89; 95%CI, 1.45 to 2.47) and DSS (HR: 2.07; 95%CI, 1.60 to 2.68).

## Discussion

Nearly 170,000 new cases of laryngeal cancer occur worldwide each year, and more than 90,000 people die of it [30]. The incidence of hypopharyngeal cancer is about a quarter of that of laryngeal cancer.

Early studies found that the incidence of positive cervical lymph node dissection of LSCC was 32.9%, and the incidence of locally advanced tumors was 70.8% [31]. Cervical metastases of HPSCC usually occur more frequently in HPSCC than in LSCC, and 80% HPSCC patients undergoing initial surgery and neck dissection suffer from cervical metastases [14, 21]. Local lymph node metastasis could increase tumor recurrence and mortality. Thus, a staging system is needed to stratify patients, and then possibly determine which patients are more suitable for aggressive treatment.

The current TNM staging was subdivided into refined grades, which allocated one positive lymph node ≤3 cm with ENE to the N2a category, and one positive Lymph node > 3 cm with ENE or any two or more positive lymph nodes with ENE to the N3b category [2]. However, the N staging does not involve the metastatic lymph node burden as an important prognostic factor in LHSCC [32], so it may underestimate the cumulative effect of burden escalation of metastatic lymph nodes [32].

Since metastatic lymph node burden is one of the most important prognostic indicators of head and neck cancers [4, 32–34], and can increase the recurrence rate and mortality [33, 35], a variety of features of lymph nodes as prognostic indicators for head and neck cancer, were investigated thoroughly. As for LHSCC, extracapsular spread (ECS) [35], number of positive lymph nodes [12, 13, 32], and lymph node yield [18, 36, 37] were reported. In recent years, several studies suggested that LNR or LND was an independent risk factor for patients with head and neck cancers [4, 33, 37–39], including LHSCC [13–16, 18, 19, 21, 22], while some other studies found no significant relationship between survival and the LNR or LND for LHSCC [12, 40]. As a result, there is no consensus on whether LNR as a significant prognostic factor complements the TNM staging system.

The systematic review and meta-analysis suggested that high LNR values reflected significantly short OS, DSS, and DFS in LHSCC patients undergoing surgery with or without neoadjuvant therapy. The subgroup analysis by region or tumor site also proved that a LNR value was related to poor survival. Besides, the analysis of only high-quality studies obtained similar results. Although publication bias might exist in the studies included, the results adjusted by the trim and fill method demonstrated the bias made no difference to the final results. We found similar results via the sensitivity analysis.

Some studies have found that the positive lymph nodes is also an important prognostic indicator in patients suffering from LHSCC, and more positive lymph nodes predicted an increased mortality rate [12, 32]. Since the number of positive lymph nodes produced is influenced by the technique of neck dissection selected, the low value may mislead practitioners into making the wrong diagnosis of the actual disease [11]. Besides, some believed that positive lymph nodes might be superior to LNR as a significant predictor for head and neck cancers, such as LHSCC [3]. In the multicenter study based on the SEER database, data on the type of neck dissection, the protocol of pathologic review of dissection, recurrence rate, the adjuvant chemotherapy, and ECS were unavailable. Similar limitations also existed in two studies [27, 28] included in this meta-analysis.

Radical, modified radical, and selective neck dissection are recommended for the removal of the tumors [1]. Previous studies considered lymph nodes yield might be associated with the survival of LHSCC [36, 41], but the lymph node yield is greatly influenced by the type of cervical lymph node dissection, and there is no universally accepted threshold up to now. Besides, lymph node hypertrophy occurring differently among individuals makes the lymph nodes yield less reliable [11]. As a result, lymph node yield may not be a good indicator of the impact of positive lymph nodes on survival.

LNR is a better prognostic measurement, for it makes a combination of information on the burden of regionally metastatic disease and type of neck dissection, so it integrates the advantages of these two parameters while bypassing their disadvantages.

In this review, the cutoff values were calculated through different methods, and the lowest significant LNR value was 0.044, which is close to the lowest value of 0.025 in oral cavity cancer reported by Huang et al [11]

To the best of our knowledge, this article is the first one that conducts a meta-analysis by taking LNR as a significant prognostic indicator for LHSCC. The pooled results suggested that LNR was an independent prognosticator of survival in patients with LHSCC, and might be able to group patients into different levels through the significance of adjuvant therapy.

There are some inherent limitations to this study. Firstly, the data of most studies were collected retrospectively. Thus, some important clinical information generally had low quality, which might render the survival results limited, such as ECS, adjuvant chemotherapy protocol and patient adherence. Additionally, the acquisition of clinicopathologic information might not be acceptable. On the other hand, long-term follow-up information was available for retrospective studies. Secondly, some studies did not adjust for all the significant factors in the multivariate analysis, such as whether to receive adjuvant chemotherapy. Moreover, most eligible studies identified LNR cutoff points through the analysis of time-dependent receiver operating characteristic curves which was based on the so-called optimal cut-point and might overestimate the effect of prognosis [42]. Thirdly, one study [12] included in the review that found LNR was not associated with survival did not report the negative adjusted HR values, and all studies included in the quantitative synthesis yielded positive results. In order to make the results more robust and reliable, we conducted a sensitivity analysis that synthesized high-quality studies and obtained similar results. Fourthly, we grouped all LHSCC to review, which may bias the accuracy of the results. But two studies [12, 18] exploring the prognostic value of LNR in LHSCC demonstrated that tumor site, whether laryngeal or hypopharyngeal, had no significant on OS, DSS and DFS. Besides, we analyzed the LSCC data separately from the HPSCC, and the subgroup analysis results was similar with the overall. Finally, the sample size of eligible studies was small, resulting in publication bias because positive results from small sample size studies are more likely to be published.

## Conclusion

This review indicates that increased high LNR values are related to poor OS, DSS, and DFS in patients with LHSCC undergoing surgery with or without neoadjuvant therapy. Nevertheless, if LNR can serve as a supplement to TNM staging and improve the accuracy of tumor staging and the cutoff values we have proposed are valid, more large samples and high-quality studies are needed to verify its reliability in the future.

## Supplementary information

**Additional file 1: Table S1**. Queries in PubMed.

**Additional file 2: Table S2.** Queries in Embase.

**Additional file 3: Table S3.** Queries in Cochrane.

**Additional file 4: Table S4.** Studies excluded in full text screening.

**Additional file 5: Figure S1.** Filled funnel plot in DSS outcome.

**Additional file 6: Figure S2.** Filled funnel plot in OS outcome.

## Data Availability

Not applicable.
