# Supplementary material for: Prognostic value of lymph node ratio in laryngeal and hypopharyngeal squamous cell carcinoma: a systematic review and meta-analysis
Source: J Otolaryngol Head Neck Surg. 2020 May 29;49:31. doi: 10.1186/s40463-020-00421-w (PMC7257235; doi:10.1186/s40463-020-00421-w)
Supplement: Supplementary file 1 — Additional file 1: Table S1. Queries in PubMed. [file 40463_2020_421_MOESM1_ESM.docx]

Table S1 Queries in PubMed

| **Search** | **Query** | **Items found** |
| --- | --- | --- |
| #31 | Search ((((("Laryngeal Neoplasms"[Mesh]) OR ((((laryngeal) OR Larynx)) AND (((cancer?) OR neoplasm?) OR carcinoma?)))) OR (("Hypopharyngeal Neoplasms"[Mesh]) OR ((Hypopharyngeal Neoplasm?) OR Hypopharyngeal cancer?)))) AND (((("Lymph Nodes"[Mesh]) OR nodal)) AND ((ratio) OR density)) | 174 |
