# Supplementary material for: Prognostic value of lymph node ratio in laryngeal and hypopharyngeal squamous cell carcinoma: a systematic review and meta-analysis
Source: J Otolaryngol Head Neck Surg. 2020 May 29;49:31. doi: 10.1186/s40463-020-00421-w (PMC7257235; doi:10.1186/s40463-020-00421-w)
Supplement: Supplementary file 2 — Additional file 2: Table S2. Queries in Embase. [file 40463_2020_421_MOESM2_ESM.docx]

**Table S2 Queries in Embase**

| Search | Queries | Items found |
| --- | --- | --- |
| 1 | "laryngeal cancer".ab,kw,ti. | 6823 |
| 2 | "laryngeal carcinoma".ab,kw,ti. | 3864 |
| 3 | "laryngeal squamous cell carcinoma".ab,kw,ti. | 2097 |
| 4 | exp larynx cancer/ | 20185 |
| 5 | exp larynx carcinoma/ | 10250 |
| 6 | exp larynx squamous cell carcinoma/ | 1731 |
| 7 | "larynx cancer".ab,kw,ti. | 1125 |
| 8 | "larynx carcinoma".ab,kw,ti. | 491 |
| 9 | "larynx squamous cell carcinoma".ab,kw,ti. | 74 |
| 10 | 1 or 2 or 3 or 4 or 5 or 6 or 7 or 8 or 9 | 23877 |
| 11 | exp hypopharynx cancer/ | 4982 |
| 12 | "hypopharyngeal cancer".ab,kw,ti. | 1448 |
| 13 | exp hypopharynx carcinoma/ | 2260 |
| 14 | "hypopharynx carcinoma".ab,kw,ti. | 90 |
| 15 | "hypopharynx cancer".ab,kw,ti. | 150 |
| 16 | "hypopharyngeal carcinoma".ab,kw,ti. | 821 |
| 17 | exp hypopharynx squamous cell carcinoma/ | 351 |
| 18 | "hypopharynx squamous cell carcinoma".ab,kw,ti. | 31 |
| 19 | "hypopharyngeal squamous cell carcinoma".ab,kw,ti. | 442 |
| 20 | 11 or 12 or 13 or 14 or 15 or 16 or 17 or 18 or 19 | 5936 |
| 21 | exp lymph node/ | 165146 |
| 22 | "lymph node".ab,kw,ti. | 192414 |
| 23 | nodal.ab,kw,ti. | 66830 |
| 24 | ratio.ab,kw,ti. | 1309280 |
| 25 | density.ab,kw,ti. | 661751 |
| 26 | 21 or 22 or 23 | 324785 |
| 27 | 24 or 25 | 1915032 |
| 28 | 26 and 27 | 26284 |
| 29 | "lymph node ratio".ab,kw,ti. | 1772 |
| 30 | "lymph node density".ab,kw,ti. | 255 |
| 31 | 28 or 29 or 30 | 26284 |
| 32 | 10 or 20 | 27039 |
| 33 | 31 and 32 | 260 |
