# Supplementary material for: Prognostic value of lymph node ratio in laryngeal and hypopharyngeal squamous cell carcinoma: a systematic review and meta-analysis
Source: J Otolaryngol Head Neck Surg. 2020 May 29;49:31. doi: 10.1186/s40463-020-00421-w (PMC7257235; doi:10.1186/s40463-020-00421-w)
Supplement: Supplementary file 3 — Additional file 3: Table S3. Queries in Cochrane. [file 40463_2020_421_MOESM3_ESM.docx]

**Table S3 Queries in Cochrane**

ID Search

#1 MeSH descriptor: [Hypopharyngeal Neoplasms] explode all trees

#2 "hypopharyngeal cancer?":ab,ti,kw

#3 "hypopharyngeal carcinoma?":ab,ti,kw

#4 "hypopharyngeal Neoplasm?":ab,ti,kw

#5 "hypopharynx cancer?":ab,ti,kw

#6 "hypopharynx carcinoma?":ab,ti,kw

#7 "hypopharynx Neoplasm?":ab,ti,kw

#8 "hypopharyngeal squamous cell carcinoma":ab,ti,kw

#9 "hypopharynx squamous cell carcinoma":ab,ti,kw

#10 #1 OR #2 OR #3 OR #4 OR #5 OR #6 OR #7 OR #8 OR #9

#11 MeSH descriptor: [Laryngeal Neoplasms] explode all trees

#12 "laryngeal cancer?":ab,ti,kw

#13 "laryngeal carcinoma?":ab,ti,kw

#14 "laryngeal Neoplasm?":ab,ti,kw

#15 "larynx cancer?":ab,ti,kw

#16 "larynx carcinoma?":ab,ti,kw

#17 "larynx Neoplasm?":ab,ti,kw

#18 "laryngeal squamous cell carcinoma":ab,ti,kw

#19 "larynx squamous cell carcinoma":ab,ti,kw

#20 #11 OR #12 OR #13 OR #14 OR #15 OR #16 OR #17 OR #18 OR #19

#21 #10 OR #20

#22 MeSH descriptor: [Lymph Nodes] explode all trees

#23 nodal:ab,ti,kw

#24 "lymph node?":ab,ti,kw

#25 #23 OR #24

#26 density:ab,ti,kw

#27 ratio:ab,ti,kw

#28 #26 OR #27

#29 #25 AND #28

#30 "lymph node? ratio":ab,ti,kw

#31 "lymph node? density":ab,ti,kw

#32 "nodal ratio":ab,ti,kw

#33 "nodal density":ab,ti,kw

#34 #29 OR #30 OR #31 OR #32 OR #33

#35 #21 AND #34
