# Supplementary material for: Prognostic value of lymph node ratio in laryngeal and hypopharyngeal squamous cell carcinoma: a systematic review and meta-analysis
Source: J Otolaryngol Head Neck Surg. 2020 May 29;49:31. doi: 10.1186/s40463-020-00421-w (PMC7257235; doi:10.1186/s40463-020-00421-w)
Supplement: Supplementary file 4 — Additional file 4: Table S4. Studies excluded in full text screening. [file 40463_2020_421_MOESM4_ESM.docx]

**Table S2 Studies excluded in full text screening**

| **Author** | **Year** | **Title** | **Reasons for exclusion** |
| --- | --- | --- | --- |
| Saha | 2014 | Impact of lymph node ratio on survival in advanced head and neck cancer: National Cancer Data Base (NCDB) | Cannot get full text |
| Hua | 2014 | Prognostic significance of the number of positive lymph nodes, number of involved regions and metastatic lymph node ratio in hypopharyngeal cancer | Duplicate of "Hua et al. 2015" |
| Shi | 2017 | Development of comprehensive nomograms for evaluating overall and cancer-specific survival of laryngeal squamous cell carcinoma patients treated with neck dissection | The same population as wang et al. 2014 |
| Sano | 2018 | Lymph node ratio as a prognostic factor for survival in patients with head and neck squamous cell carcinoma. | Wrong population |
| Jacobi | 2018 | Prognostic value of the lymph node ratio in oropharyngeal carcinoma stratified for HPV-status | Wrong population |
| Trifiletti | 2017 | Beyond Positive Margins and Extracapsular Extension: Evaluating the Utilization and Clinical Impact of Postoperative Chemoradiotherapy in Resected Locally Advanced Head and Neck Cancer | Wrong population |
| Yildiz | 2016 | Which is the most suitable lymph node predictor for overall survival after primary surgery of head and neck cancer: pN, the number or the ratio of positive lymph nodes, or log odds? | Wrong population |
| Divi | 2016 | Lymph node count from neck dissection predicts mortality in head and neck cancer | Wrong population |
| Dequanter | 2016 | Prognostic value of lymph node ratio for locoregional failure in patients with advanced head and neck cancers | Wrong population |
| Prabhu | 2015 | Lymph node ratio influence on risk of head and neck cancer locoregional recurrence after initial surgical resection: Implications for adjuvant therapy | Wrong population |
| Chen | 2015 | Lymph node ratio as a prognostic factor in head and neck cancer patients | Wrong population |
| Reinisch | 2014 | Is lymph-node ratio a superior predictor than lymph node status for recurrence-free and overall survival in patients with head and neck squamous cell carcinoma? | Wrong population |
| Chufal | 2006 | Analysis of prognostic variables among patients with locally advanced head and neck cancer treated with late chemo-intensification protocol: impact of nodal density and total tumor volume | Wrong population |
| Zumsteg | 2019 | Quantitative lymph node burden as a 'very-high-risk' factor identifying head and neck cancer patients benefiting from postoperative chemoradiation | wrong predictor |
| Cayonu | 2019 | Lymph node yield and lymph node density for elective level II-IV neck dissections in laryngeal squamous cell carcinoma patients | wrong predictor |
| Tassone | 2018 | Association of Positive Initial Margins With Survival Among Patients With Squamous Cell Carcinoma Treated With Total Laryngectom | wrong predictor |
| Ho | 2018 | Association of Quantitative Metastatic Lymph Node Burden With Survival in Hypopharyngeal and Laryngeal Cancer | wrong predictor |
| Wachters | 2017 | Phosphorylated FADD is not prognostic for local control in T1-T2 supraglottic laryngeal carcinoma treated with radiotherapy. | wrong predictor |
| Massa | 2017 | Survival after refusal of surgical treatment for locally advanced laryngeal cancer | wrong predictor |
| Fletcher | 2017 | Prognostic indicators and survival in salvage surgery for laryngeal cancer | wrong predictor |
| Divi | 2016 | Establishing quality indicators for neck dissection: Correlating the number of lymph nodes with oncologic outcomes (NRG Oncology RTOG 9501 and RTOG 0234) | wrong predictor |
| Bottcher | 2016 | Nodal yield of neck dissections and influence on outcome in laryngectomized patients | wrong predictor |
| Oosterkamp | 2006 | Predictive value of lymph node metastases and extracapsular extension for the risk of distant metastases in laryngeal carcinoma | wrong predictor |
