# Supplementary figures and images for: Prognostic value of lymph node ratio in laryngeal and hypopharyngeal squamous cell carcinoma: a systematic review and meta-analysis
Source: J Otolaryngol Head Neck Surg. 2020 May 29;49:31. doi: 10.1186/s40463-020-00421-w (PMC7257235; doi:10.1186/s40463-020-00421-w)

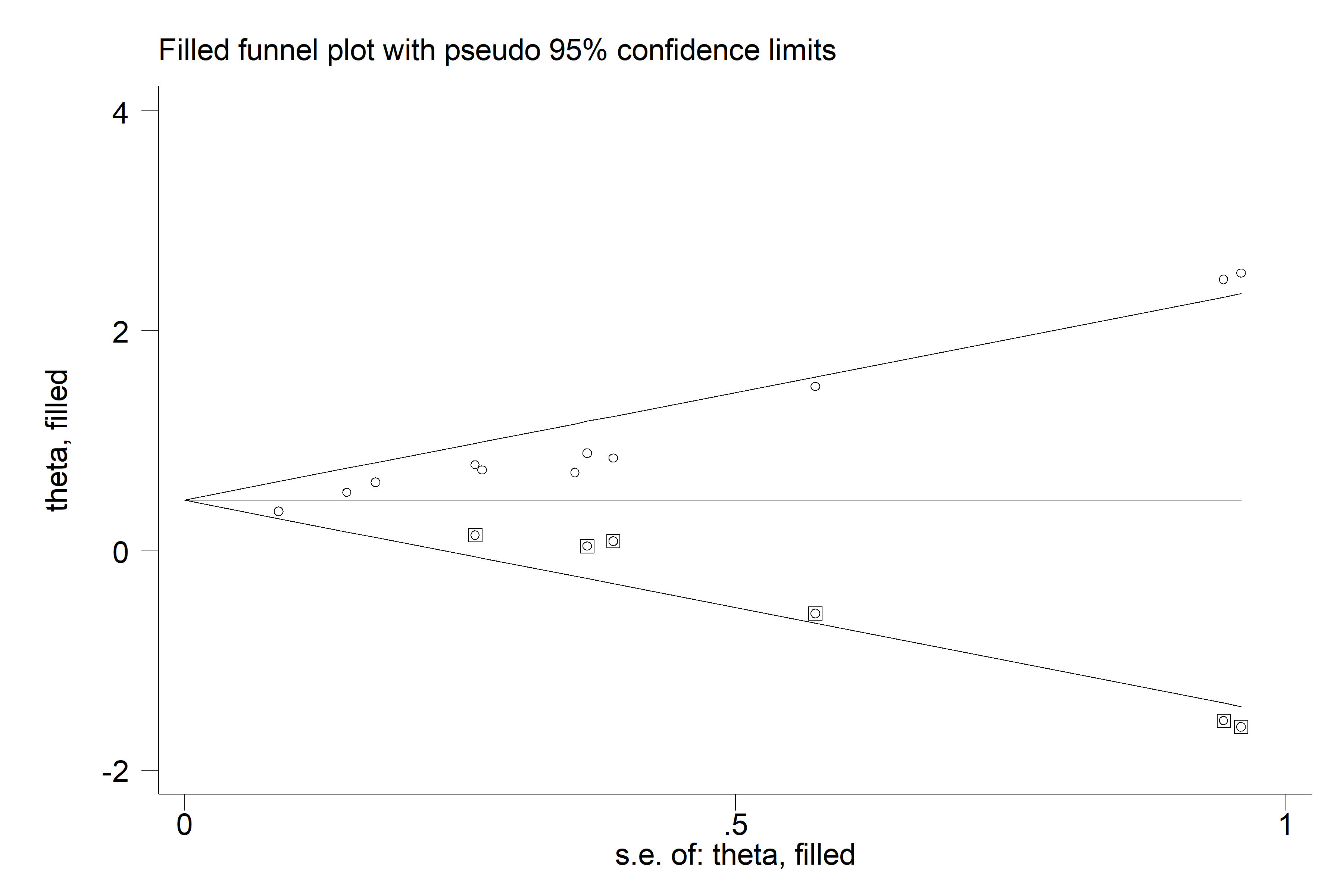

Supplement: Supplementary file 5 — Additional file 5: Figure S1. Filled funnel plot in DSS outcome. [file 40463_2020_421_MOESM5_ESM.tif]

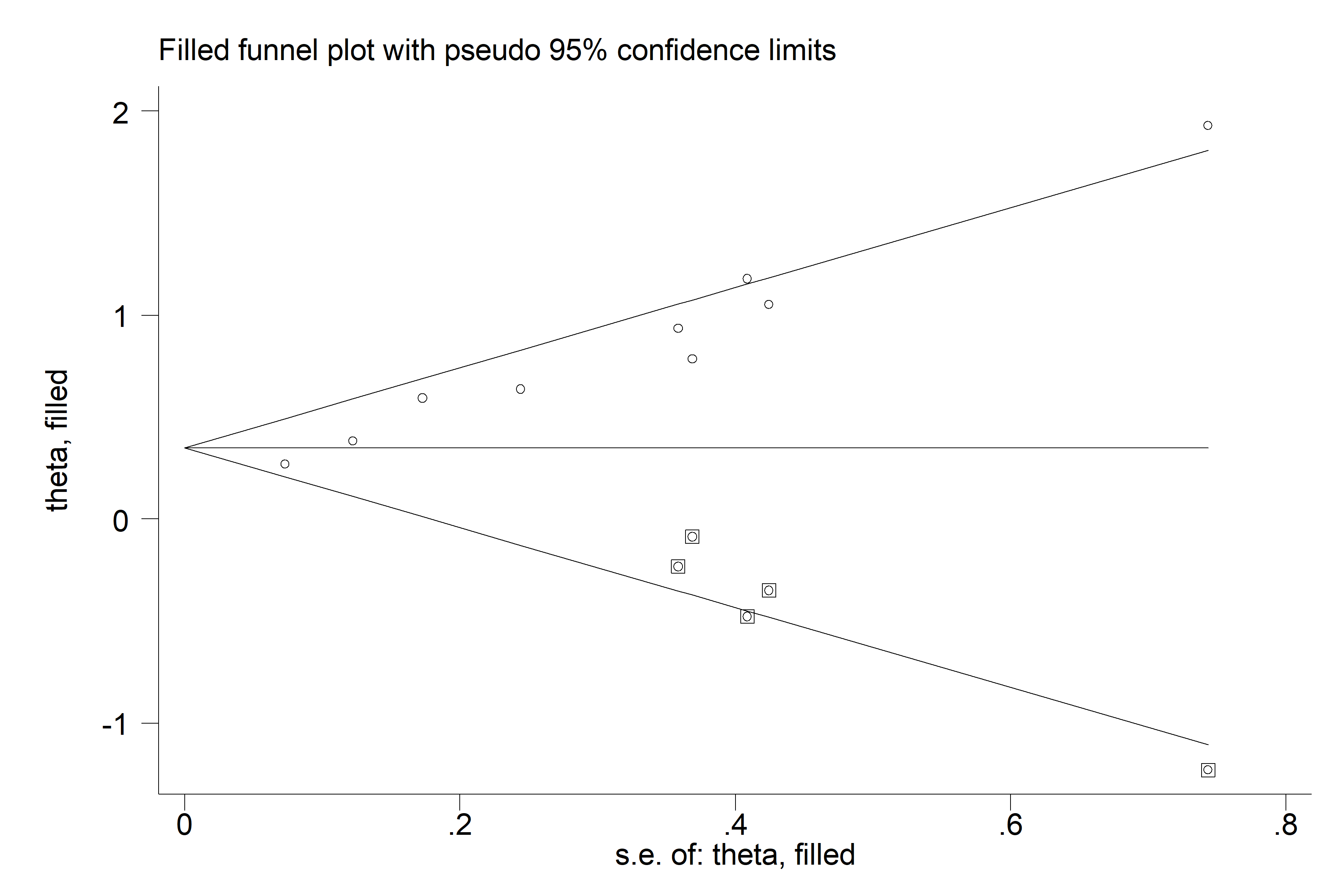

Supplement: Supplementary file 6 — Additional file 6: Figure S2. Filled funnel plot in OS outcome. [file 40463_2020_421_MOESM6_ESM.tif]
